# The Royal British Nurses' Association

**Published:** 1893-06-24

**Authors:** 


### THE ROYAL BRITISH NURSES' ASSOCIATION.

We print in another column the full text of the Charter which has been granted to the Royal British Nurses' Association. The perusal of this document will render it abundantly clear why the authorities of the Association declined to give the information asked for by Dr. Troutbeck and Mr. Henry C. Burdett in their letters to the *Times*, which we reprinted in our issue of the 3rd inst. Now that we are in possession of the information which the Association then withheld, we are able to appreciate the discreetness of their silence. The Charter which has been granted does not empower them to maintain a general register of trained nurses; still less does it enable them to institute "a system of registering nurses analagous to that enforced by law for many years past for medical men."

The purposes for which the Association has, in fact, been incorporated are the following:—

1. The founding and maintenance of schemes for the benefit of nurses in the practice of their profession, and in times of adversity, sickness, and old age.
2. The maintenance of an office or offices for supplying information to persons seeking for nurses, and to persons seeking for employment as nurses.
3. The maintenance and publication of a list of persons who may have applied to the corporation to have their names entered therein as nurses, and whom the corporation may think fit to enter therein from time to time, coupled with such information about each person so entered as to the corporation may from time to time seem desirable.
4. The promotion of conferences, public meetings, and lectures in connection with the general work of the corporation.
5. The doing anything incidental or conducive to carrying into effect the foregoing purposes.

It is scarcely necessary to repeat what we recently called attention to, that the opposition which was offered to the application of the Royal British Nurses' Association was directed only to the question of registration. So far as their benevolent and charitable objects are concerned, we are able disinterestedly to congratulate them on the distinction which they have obtained. But, leaving these out of account, what is it which is now to be substituted for the general register of nurses? The Association are empowered to establish and carry on a Registry Office for Nurses, and to publish a list of the nurses whom they are prepared to recommend. To call such a list a register would be as absurd as it would be to dignify by that name a list of governesses or tutors kept by a firm of education agents. If the Association think that the fact of their having been incorporated for such objects as these is matter for congratulation, on this ground also we are willing to congratulate them.

